# Supplementary material for: Splice-Junction-Based Mapping of Alternative Isoforms in the Human Proteome
Source: Cell Rep. Author manuscript; Available in PMC 2020 Jan 15. (PMC6961840; doi:10.1016/j.celrep.2019.11.026)
Supplement: 2 [file NIHMS1546469-supplement-2.zip › DF1/PXD009737/TestisLysC/TestisLysC_19_KIAA1210_NLQVIVEPK.pdf]

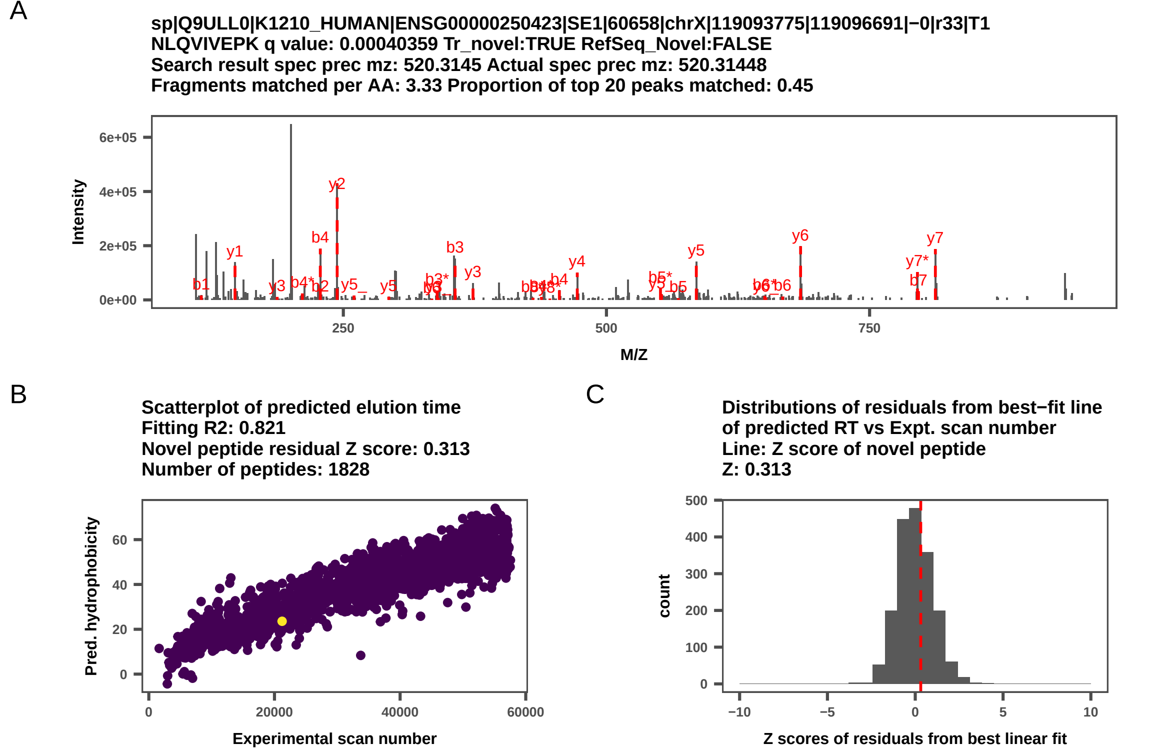
A
sp|Q9ULL0|K1210_HUMAN|ENSG00000250423|SE1|60658|chrX|119093775|119096691|−0|r33|T1
NLQVIVEPK q value: 0.00040359 Tr_novel:TRUE RefSeq_Novel:FALSE
Search result spec prec mz: 520.3145 Actual spec prec mz: 520.31448
Fragments matched per AA: 3.33 Proportion of top 20 peaks matched: 0.45
Intensity
6e+05
4e+05
2e+05
0e+00
y1
y2
b4
b3
y3
y4
y5
y6
y7
y7*
b7
b1
y3
b4*
b2
y5_
y5
b3*
b5*
b5
b6*
b6
b4
250
500
750
M/Z
B
Scatterplot of predicted elution time
Fitting R2: 0.821
Novel peptide residual Z score: 0.313
Number of peptides: 1828
Pred. hydrophobicity
60
40
20
0
0
20000
40000
60000
Experimental scan number
C
Distributions of residuals from best−fit line
of predicted RT vs Expt. scan number
Line: Z score of novel peptide
Z: 0.313
count
500
400
300
200
100
0
−10
−5
0
5
10
Z scores of residuals from best linear fit
